# Nurses' Pension Funds

**Published:** 1887-02-26

**Authors:** W. Snape

**Affiliations:** 109, Sussex Road, Holloway, N.


Mr. R. G. Salmond neglects to state in his letter, *re* above, that the rules of the Trained Nurses' Annuity Fund, of which he is secretary, exclude nurses under the age of 50, and require 15 years' active service, three of which must have been passed in a public hospital or training institution. Is there not some philanthropic person who will start a fund the object of which would be to benefit nurses of over five or seven years' service whose health has been undermined by the constant and watchful care of these trained friends, or could not Mr. Salmond take steps to let the nurses themselves know more of the Society of which he writes? Hoping you will take the matter up, and thus benefit a most deserving praiseworthy class. W. Snape.

